# Three-year changes in ocular biometric components in children with amblyopia

**DOI:** 10.1186/s12886-025-03895-2

**Published:** 2025-02-10

**Authors:** Masoud Khorrami-Nejad, Roqayeh Aliyari, Ali Nouraeinejad, Mohsen Heirani, Mohammad Hassan Emamian, Hassan Hashemi, Akbar Fotouhi

**Affiliations:** 1 School of Rehabilitation, Tehran University of Medical Sciences, Tehran, Iran; 2 Ophthalmic Epidemiology Research Center, Shahroud University of Medical Sciences, Shahroud, Iran; 3 Noor Ophthalmology Research Center, Noor Eye Hospital, Tehran, Iran; 4 Department of Epidemiology and Biostatistics, School of Public Health, Tehran University of Medical Sciences, Tehran, Iran; 5 Hafte Tir Square, Shahroud University of Medical Sciences and Health Services, Shahroud, 3614773955 Iran; 6 Center for Health Related Social and Behavioral Sciences Research, Shahroud University of Medical Sciences, Shahroud, Iran

**Keywords:** Ocular biometry, Amblyopia, Axial length, Keratometry, Refraction

## Abstract

**Background:**

Amblyopia is associated with structural differences in ocular biometrics, but existing studies often lack long-term follow-ups. This study compares three-year changes in ocular biometric components in amblyopic and non-amblyopic eyes.

**Methods:**

In this prospective cohort, study data were collected in 2015 and 2018 in Shahroud, northeast Iran. The sample comprised 4968 primary students (9935 eyes), including 4931 non-amblyopic students (9893 eyes) and 37 students with amblyopia (42 eyes). Axial length, keratometry, central corneal thickness, lens thickness, pupil diameter, anterior chamber depth, lens power and vitreous chamber depth were measured using Lenstar LS900. Multilevel mixed-effects regression models were used to determine any association between variables.

**Results:**

The mean age of children without and with amblyopia at baseline were 9.7 ± 1.7 and 9.9 ± 1.5 years, respectively. The mean axial elongation in amblyopic and non-amblyopic groups over three years was 0.37 (95% CI: 0.34–0.40) and 0.33 mm (95% CI: 0.31–0.34), respectively (*p* < 0.001). Amblyopic eyes showed greater axial elongation than non-amblyopic eyes (*p* < 0.001). Anterior chamber depth was constant in amblyopic eyes while it decreased in non-amblyopic eyes by 0.13 mm. Vitreous chamber depth increased significantly in both groups, with a greater increase observed in the amblyopic eyes (0.36 vs. 0.28 mm) (*p* < 0.001). Flat and steep keratometry increased significantly in amblyopic eyes (*p* < 0.001), while it was constant in non-amblyopic eyes. Lens power decreased significantly in both groups (*p* < 0.001). The increase in the axial length and vitreous chamber depth in the amblyopic eye was greater than in the non-amblyopic eyes (*p* < 0.001). The spherical equivalent change in both groups was − 0.31 D.

**Conclusions:**

Amblyopia was associated with significant changes in key biometric parameters, offering valuable insight into the structural alterations underlying the condition.

## Introduction

 Visual system development in early childhood involves both structural and functional maturation of the eyes and visual neural pathways [1, 2]. If there is a disruption in normal visual development during this sensitive period of neuroplasticity, it can lead to amblyopia, a neurodevelopmental disorder characterized by reduced best-corrected visual acuity without structural ocular or visual pathway defects [3]. Amblyopia is typically unilateral and associated with impaired or absent stereopsis, although both eyes may be affected [4–6]. 

Amblyopia represents one of the most prevalent causes of unilateral visual impairment among children worldwide, with an estimated prevalence ranging from 0.51 to 3.67% in the general population [7]. Amblyopic children experience abnormal visual development, often unaware of their monocular visual deficit. Early diagnosis and intervention can preserve normal vision development [4, 8, 9]. 

Abnormal refraction constitutes a principal risk factor for amblyopia development. Timely detection and management of refractive errors is a broadly accepted clinical approach to reduce the risk of refractive and strabismic amblyopia subtypes. In addition to normal refraction, proper ocular growth is critical for optimal visual function [10]. Continuous alterations in the ocular refractive system throughout life play a vital role in normal visual development [11, 12]. 

Several previous studies have underscored the importance of understanding ocular structural changes in amblyopia. For example, Cass and Tromans found differences in lens thickness, anterior chamber depth, and axial length between amblyopic and fellow eyes, suggesting amblyopia impacts ocular development [13]. Additionally, Demircan et al. noted differences in corneal thickness and curvature between amblyopic and normal eyes [14]. These studies demonstrate that differences in ocular biometrics exist in amblyopia and likely contribute to its pathogenesis. Numerous studies have attempted to correlate refraction with ocular biometric parameters [15, 16]. During infancy, the axial length, anterior and vitreous chamber depths, and crystalline lens radius all increase. In this period, the refractive error becomes less hyperopic and then remains relatively stable [17]. The adjustment of axial growth in response to initial refractive error plays a crucial role in decreasing refractive error during the emmetropization process [12]. 

Ocular biometric parameters change continually after birth, and non-emmetropic refractive errors arise from mismatches in these measurements [18]. While studies have reported refractive and biometric findings in children with amblyopia, most have utilized relatively small sample sizes. Furthermore, the majority of these studies did not specify the amblyopia subtype. Ocular biometry research has provided an enhanced understanding of the etiology of refractive errors. Thus, prospective cohort studies are essential to expand knowledge related to amblyopia. However, few relevant studies exist, and most either failed to recruit adequately large samples, conducted only a single follow-up, or were not population-based [13, 19–22]. It is crucial to use cohort studies with modern ophthalmic and biometric tools to assess long-term changes in refraction and ocular biometry in amblyopic versus non-amblyopic eyes. Understanding differences in ocular growth patterns could enable earlier detection of amblyopia risk, tailored treatment plans, improved monitoring of outcomes, and deeper insights into the condition’s pathophysiology. To the best of our knowledge, no previous studies have examined longitudinal changes in these ocular parameters using a sufficiently large sample over an extended follow-up period. Given the importance of refractive and related ocular biometric measures in amblyopia development, the present study aimed to assess changes in refraction and associated biometric parameters in amblyopic children over a three-year follow-up period.

## Methods

The Shahroud Schoolchildren Eye Cohort Study (SSCECS), which collected data in 2015 and 2018 (3-years follow-up), was conducted in Shahroud, north-eastern Iran. [23] In 2015 (the first phase), 6624 students were invited, and 5620 students (84.8%) participated in the study. The second phase of the study was conducted in 2018, and 5292 (94.2%) of the first phase participants participated in the second phase. To better evaluate the rural population, all primary school children in rural areas (1214 children) were invited to join. While in urban areas, children were chosen by random cluster sampling, and classrooms were picked as clusters. The details of this cohort profile were previously described by Emamian et al. [23] The study population included all students who participated in both phases of the SSCECS (2015 and 2018) and students with a history of ocular surgery, pathological findings, or developmental delay were excluded from the analysis.

The study adhered to the tenets of the Declaration of Helsinki during all examination phases. The examination procedures were thoroughly explained to all student participants in both assessment periods. The research protocol received approval from the Ethics Committee of Shahroud University of Medical Sciences. Signed written informed consent was obtained from parents or legal guardians of all participants. Verbal assent was additionally acquired from each student.

The diagnosis of unilateral amblyopia was based on ≥ 2 line interocular difference in best-corrected visual acuity with 0.2 logMAR or worse in the amblyopic eye, along with at least one of the following amblyogenic factors: (1) anisometropia (difference in myopia, hyperopia, and astigmatism equal or more than 3.00 D, 1.00D and 1.50 D, respectively); (2) strabismus; or (3) a combination of both [24]. The amblyopic cohort comprised 42 eyes, including 39 with refractive, strabismic, or combined mechanisms and 3 with form-deprivation amblyopia.

As per the Amblyopia Treatment Studies (ATS) guidelines [25, 26], the standard protocols for passive occlusion therapy were outlined as follows: children with severe amblyopia (CDVA worse than 20/100 or 0.7 logMAR) were advised to patch the fellow eye for 6 h daily. Meanwhile, children with moderate amblyopia (CDVA ranging between 20/80 and 20/40 or 0.6–0.3 logMAR) and those with mild amblyopia (CDVA between 20/40 and 20/30 or 0.3–0.2 logMAR) were instructed to patch the fellow eye for 2 h per day, without incorporating additional visual training [8, 27]. 

All students and their parents or guardians in the selected classrooms received questionnaires to obtain contact details, ophthalmic history, parental employment, insurance coverage, students’ daily activities on school days and weekends, and nutritional status. After coordinating with schools, an examination day was scheduled for each classroom. UDVA was first measured using the Snellen chart Projector (CP-770; Nidek, Japan). Then, non-cycloplegic autorefraction was performed with the Nidek ARK-510 A auto-refractor-keratometer (Nidek Co. Ltd, Gammagori, Aichi, Japan), followed by dry retinoscopy to refine and record objective refraction. Subjective refraction was subsequently assessed and CDVA was obtained.

Prior to cycloplegia, ocular biometric parameters were measured bilaterally using the Lenstar LS 900 optical biometer (Haag-Streit AG, Switzerland). Measured parameters included axial length, corneal curvature radii (K1, K2), central corneal thickness, lens thickness, pupil diameter, anterior chamber depth, and vitreous chamber depth [28]. Tropias and phorias were identified using the unilateral cover test (cover-uncover test) and the alternating cover test, respectively.

Cycloplegia was achieved by instilling two drops of 1% cyclopentolate in each eye at 5-minute intervals, followed by cycloplegic autorefraction and retinoscopy at least 30 min after the second drop. If necessary, a third drop was administered.

An expert optometrist diagnosed the participants with amblyopia and performed vision, refraction, and binocular vision assessments. Exam records were thoroughly checked by a senior optometrist to ensure the accuracy of the diagnoses. This included reviewing the results of the vision, refraction, and other evaluations conducted during the examination process.

A spherical equivalent (SE) of −0.50 diopters or lower was classified as myopia, while an SE of + 2.00 diopters or higher was classified as hyperopia. Emmetropia was defined as an SE between − 0.50 and 2.00 diopters. Bennett’s formula [29] was used to calculate lens power, incorporating data on cycloplegic SE, corneal power, axial length (AL), anterior chamber depth (ACD), and lens thickness (LT). Follow-up assessment in 2018 remeasured all biometric parameters using an identical methodology. Changes in ocular biometrics over the 3-year study period were analyzed and compared between amblyopic and non-amblyopic eyes. Ocular biometric changes were also investigated between amblyopic and fellow eyes, in participants who had unilateral amblyopia.

In this study, the term ‘fellow eye’ was used to refer to the non-amblyopic eye in patients with unilateral amblyopia, while ‘non-amblyopic eye’ was used to describe the normal eye in the control group to ensure clear differentiation.

### Statistical analysis

Multilevel mixed-effects models with the Residual Maximum Likelihood estimation method were used to compare amblyopic and non-amblyopic eye participant groups. The models controlled for intra-subject correlation and time dependency, with covariates including BMI, age, sex, residence place (urban or rural), time (phase 1 and phase 2), amblyopia diagnosis (yes or no), and the interaction between time and amblyopia. Dependent variables included axial length, corneal curvature radii (K1, K2), central corneal thickness, lens thickness, pupil diameter, anterior chamber depth, and vitreous chamber depth. The data were structured hierarchically with two levels: eyes (right and left) within participant and participants within time (phase 1 and phase 2); also, a random coefficient for the individual was included. A significant interaction between time and amblyopia was interpreted as the change scores between amblyopia and non-amblyopic eyes over time. A *p*-value < 0.050 was considered statistically significant. The percent changes for the ocular biometric components have been calculated by the formula 100*(value in phase 2 - value in phase 1)/value in phase 1.

## Results

Ocular biometric data and refraction results from both phases of the study were available for 4968 people, and the final analysis was performed on these participants. The mean age of participants at baseline (phase 1 of the study) was 9.7 ± 1.7 years. Of the 2,633 participants, 1,394 (53.0%) were male. The mean age of amblyopic children (*n* = 37) was 9.9 ± 1.5 years and 22 (59.5%) were male The mean body mass index (BMI) was 17.2 ± 3.3 for children without amblyopia and 17.1 ± 2.9 for those with amblyopia. Among the children without amblyopia 20.5% were rural residents, while 24.3% of children with amblyopia lived in rural areas. Five students had bilateral amblyopia, meaning a total of 42 eyes with amblyopia were available for analyses. Among the amblyopic eyes, four were diagnosed with strabismus and 17 (41%) had the best corrected visual acuity of equal to or greater than 0.3 LogMar (20/40) at the baseline phase. Low vision cases decreased to 13 (31%) at the follow-up phase.

Table 1 reports the ocular biometric components and spherical equivalent changes in the 42 amblyopic eyes and 9,893 non-amblyopic eyes. This table shows that the mean axial elongation in amblyopic and non-amblyopic groups after three years were 0.37 and 0.33 mm, respectively. The mean axial length in both groups increased significantly (*p* < 0.001), but the axial elongation in amblyopic eyes was higher than in non-amblyopic eyes (Interaction coefficient = 0.04 (CI 95%: 0.2–0.07); *p* < 0.001). The anterior chamber depth in amblyopic eyes was 3.59 mm in 2015 and 3.62 mm in 2018, respectively (*p* = 0.966). In contrast, the anterior chamber depth in non-amblyopic eyes decreased from a mean of 3.75 mm in 2015 to 3.62 mm in 2018, showing a significant reduction (*p* = 0.004). In the amblyopic and non-amblyopic eye groups, vitreous chamber depth increased significantly (*p* < 0.001), but this increase in the amblyopic eyes was higher than in the non-amblyopic eyes (Interaction coefficient = 0.08 (95% CI: 0.4–0.11; *p* < 0.001). Flat and steep keratometry measurements remained relatively stable in the non-amblyopic group (*p* = 0.988 and *p* = 0.709, respectively). However, in the amblyopic group, there was a significant increase in both flat and steep keratometry measurements (both *p* < 0.001). In both groups, pupil diameter and lens power decreased significantly after three-year (*p < 0.001*). In both study groups, the central corneal and lens thickness remained stable after three years. There was the same significant reduction (−0.31 D) of spherical equivalent in both groups (*p* < 0.001).

**Table 1:** The mean value with 95% confidence intervals (in parentheses) and three-year change of ocular biometric components and spherical equivalent in the 42 amblyopic eyes (32 unilateral and five bilateral cases) and 9,893 non-amblyopic eyes

Biometrics Attributes	Non-amblyopic eyes (*n* = 9893)				Amblyopic eyes (*n* = 42)				p**
	2015	2018	Change	p*	2015	2018	Change	p*	
Axial Length (mm)	22.99 (22.95, 23.03)	23.32 (23.26, 23.37)	0.33 (0.31, 0.34)	**< 0.001**	23.02 (22.95, 23.08)	23.39 (23.32, 23.46)	0.37 (0.34, 0.40)	**< 0.001**	**< 0.001**
Central Corneal Thickness (µm)	549.9 (548.5, 551.3)	549.9 (548.3, 551.5)	0.02 (−0.45, 0.48)	0.942	550.7 (548.2, 553.2)	550.6 (547.9, 553.2)	−0.13 (−2.09, 1.84)	0.900	0.883
Anterior Chamber Depth (mm)	3.75 (3.66, 3.84)	3.62 (3.53, 3.71)	−0.13 (−0.22, 0.04)	**0.004**	3.59 (2.43, 4.74)	3.62 (2.46, 4.77)	0.03 (−1.37, 1.43)	0.966	0.820
Lens Thickness (mm)	3.49 (3.48, 3.49)	3.49 (3.48, 3.50)	0.0 (−0.003, 0.003)	0.976	3.52 (3.49, 3.55)	3.49 (3.45, 3.52)	−0.03 (−0.07, 0.005)	0.089	0.089
Flat keratometry (diopter)	43.15 (43.09, 43.22)	43.13 (43.05, 43.20)	−0.02 (−0.05, 0.01)	0.100	41.87 (41.58, 42.15)	42.74 (42.43, 43.04)	0.87 (0.52, 1.22)	**< 0.001**	**< 0.001**
Steep keratometry (diopter)	44.04 (43.99, 44.09)	44.02 (43.97, 44.08)	−0.01 (−0.04, 0.01)	0.283	43.25 (42.91, 43.59)	43.90 (43.54, 44.25)	0.64 (0.26, 1.03)	**0.001**	**0.001**
Corneal Diameter (mm)	12.27 (12.25, 12.29)	12.30 (12.28, 12.33)	0.03 (0.02, 0.04)	**< 0.001**	12.26 (12.18, 12.34)	12.29 (12.21, 12.37)	0.03 (−0.07, 0.12)	0.570	0.960
Pupil Diameter (mm)	6.05 (6.03, 6.07)	4.18 (4.15, 4.20)	−1.87 (−1.89, −1.86)	**< 0.001**	5.98 (5.79, 6.18)	4.31 (4.10, 4.52)	−1.67 (−1.91, −1.44)	**< 0.001**	0.096
Vitreous Chamber Depth (mm)	15.93 (15.91, 15.95)	16.21 (16.19, 16.23)	0.28 (0.28, 0.29)	**< 0.001**	15.91 (15.85, 15.97)	16.27 (16.21, 16.33)	0.36 (0.32, 0.40)	**< 0.001**	**< 0.001**
Spherical Equivalent (mm)	0.81 (0.79, 0.84)	0.51 (0.48, 0.54)	−0.31 (−0.32, −0.30)	**< 0.001**	0.58 (0.44, 0.71)	0.27 (0.13, 0.42)	−0.31 (−0.45, −0.16)	**< 0.001**	0. 976
Lens Power (diopter)	21.58 (21.54, 21.62)	20.92 (20.87, 20.97)	−0.66 (−0.69, −0.63)	**< 0.001**	21.91 (21.56, 22.27)	20.82 (20.43, 21.22)	−1.09 (−1.54, −0.65)	**< 0.001**	0.055

p*: comparing two study phases (2015 and 2018) based on multilevel regression models by controlling sex, age, residence place and BMI

p**: comparing three-year changes between two study groups in multilevel regression models and by controlling sex, age, residency place and BMI

Table 2 provides ocular biometric components and spherical equivalent changes in unilateral amblyopia patients. Both groups showed increases in axial length and vitreous chamber depth, while decreases were noted in pupil diameter and lens power (*p* < 0.050). However, central corneal thickness, lens thickness, corneal diameter, and radius of curvature of the cornea (K1 and K2) remained stable over the three-year period. Lens power decreased significantly in both groups, but the decrease in non-amblyopic eyes was more prominent (−1.07 vs. −0.69 D) (*p* = 0.027).

**Table 2:** The mean value with 95% confidence intervals (in parentheses) and three-year changes of ocular biometric components and spherical equivalent in the amblyopic and fellow eyes (*n* = 32)

Biometrics Attributes	fellow eye				Amblyopic eye				p**
	2015	2018	Change	p*	2015	2018	Change	p*	
Axial Length (mm)	23.13 (22.58, 23.69)	23.54 (22.98, 24.11)	0.41 (0.33, 0.49)	**< 0.001**	23.17 (22.61, 23.72)	23.61 (23.05, 24.18)	0.45 (0.36, 0.53)	**< 0.001**	0.317
Central Corneal Thickness (µm)	539.8 (524.5, 555.2)	539.1 (523.3, 554.8)	−0.8 (−3.3, 1.8)	0.561	540.7 (525.4, 556,1)	539.7 (523.9, 555.5)	−1.0 (−3.6, 1.5)	0.432	0.795
Anterior Chamber Depth (mm)	3.58 (3.46, 3.70)	3.61 (3.49, 3.74)	0.03 (−0.004, 0.07)	0.078	3.59 (3.47, 3.71)	3.64 (3.52, 3.77)	0.05 (0.02, 0.09)	**0.003**	0.172
Lens Thickness (mm)	3.41 (3.34, 3.49)	3.40 (3.31, 3.50)	−0.01 (−0.07, 0.04)	0.664	3.44 (3.37, 3.52)	3.40 (3.31, 3.50)	−0.04 (−0.10, 0.01)	0.135	0.338
Flat keratometry (diopter)	43.56 (41.57, 45.54)	43.57 (41.52, 45.60)	0.002 (−0.28, 0.28)	0.988	42.12 (40.14, 44.11)	42.18 (40.13, 44.22)	0.053 (−0.23, 0.33)	0.712	0.181
Steep keratometry (diopter)	45.36 (43.29, 47.43)	45.40 (43.13, 47.48)	0.03 (−0.14, 0.21)	0.709	44.23 (42.16, 46.30)	44.18 (42.10, 46.27)	−0.05 (−0.22, 0.12)	0.572	0.298
Corneal Diameter (mm)	12.12 (11.95, 12.29)	12.16 (11.99, 12.33)	0.035 (−0.05, 0.12)	0.435	12.14 (11.97, 12.30)	12.14 (11.97, 12.30)	0.001 (−0.09, 0.09)	0.978	0.591
Pupil Diameter (mm)	5.99 (5.62, 6.36)	4.21 (3.78, 4.63)	−1.78 (−2.06, −1.51)	**< 0.001**	6.00 (5.63, 6.36)	4.34 (3.91, 4.76)	−1.66 (−1.93, −1.38)	**< 0.001**	0.475
Vitreous Chamber Depth (mm)	16.15 (15.60, 16.71)	16.54 (16.00, 17.11)	0.38 (0.30, 0.47)	**< 0.001**	16.15 (15.59, 16.71)	16.59 (16.01, 17.17)	0.44 (0.35, 0.53)	**< 0.001**	0.143
Spherical Equivalent (mm)	−0.53 (−2.45, 1.19)	−0.73 (−2.49, 1.02)	−0.20 (−0.55, 0.14)	0.250	−0.68 (−2.40, 1.04)	−1.06 (−2.81, 0.70)	−0.38 (−0.72, −0.03)	**0.033**	0.359
Lens Power (diopter)	21.75 (20.79, 22.71)	20.69 (19.38, 21.99)	−1.07 (−1.65, −0.48)	**< 0.001**	21.58 (20.61, 22.54)	20.89 (19.58, 22.20)	−0.69 (−1.27, −0.10)	**0.022**	**0.027**

p*: comparing two study phases (2015 and 2018) based on multilevel regression models by controlling sex, age, residence place and BMI

p**: comparing three-year changes between two study groups in multilevel regression models and by controlling sex, age, residency place and BMI

**Fig. 1:**
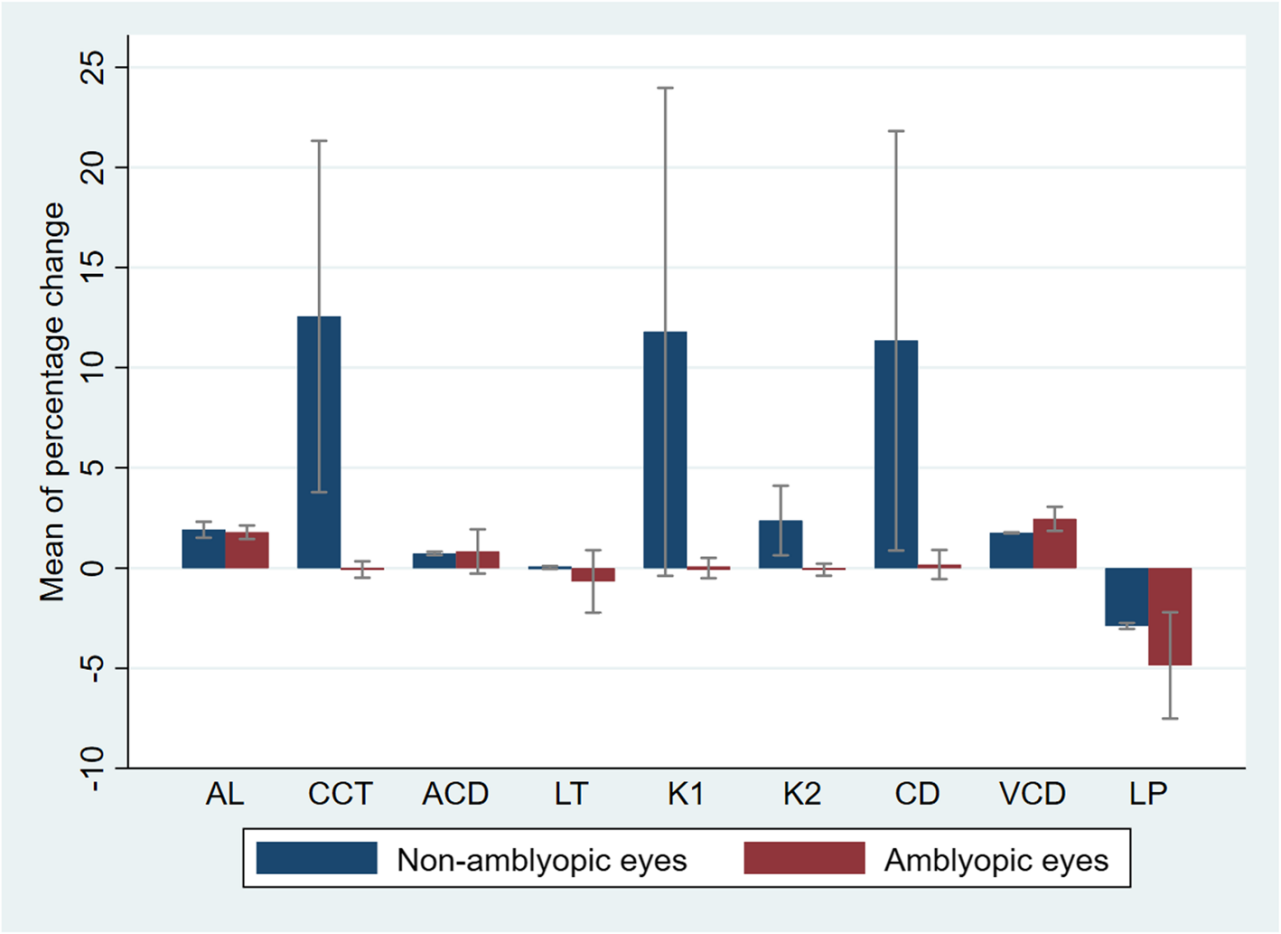
The three-year changes (in percent) of ocular biometric components in non-amblyopic and amblyopic eyes. Error bars represent 95% confidence intervals for percent changes. A positive mean change indicates greater values in phase 2 compared to phase 1 of the study AL, Axial length; CCT, central corneal thickness; ACD, anterior chamber depth; LT, lens thickness; K1, flat keratometry; K2, steep keratometry; CD, corneal diameter; VCD, vitreous chamber depth

The percent changes of ocular biometric components in non-amblyopic, and amblyopia eyes are shown in Fig. 1. This figure shows that unilateral amblyopic children exhibited the most significant changes in central corneal thickness, K1, and corneal diameter among all biometric components.

**Fig. 2:**
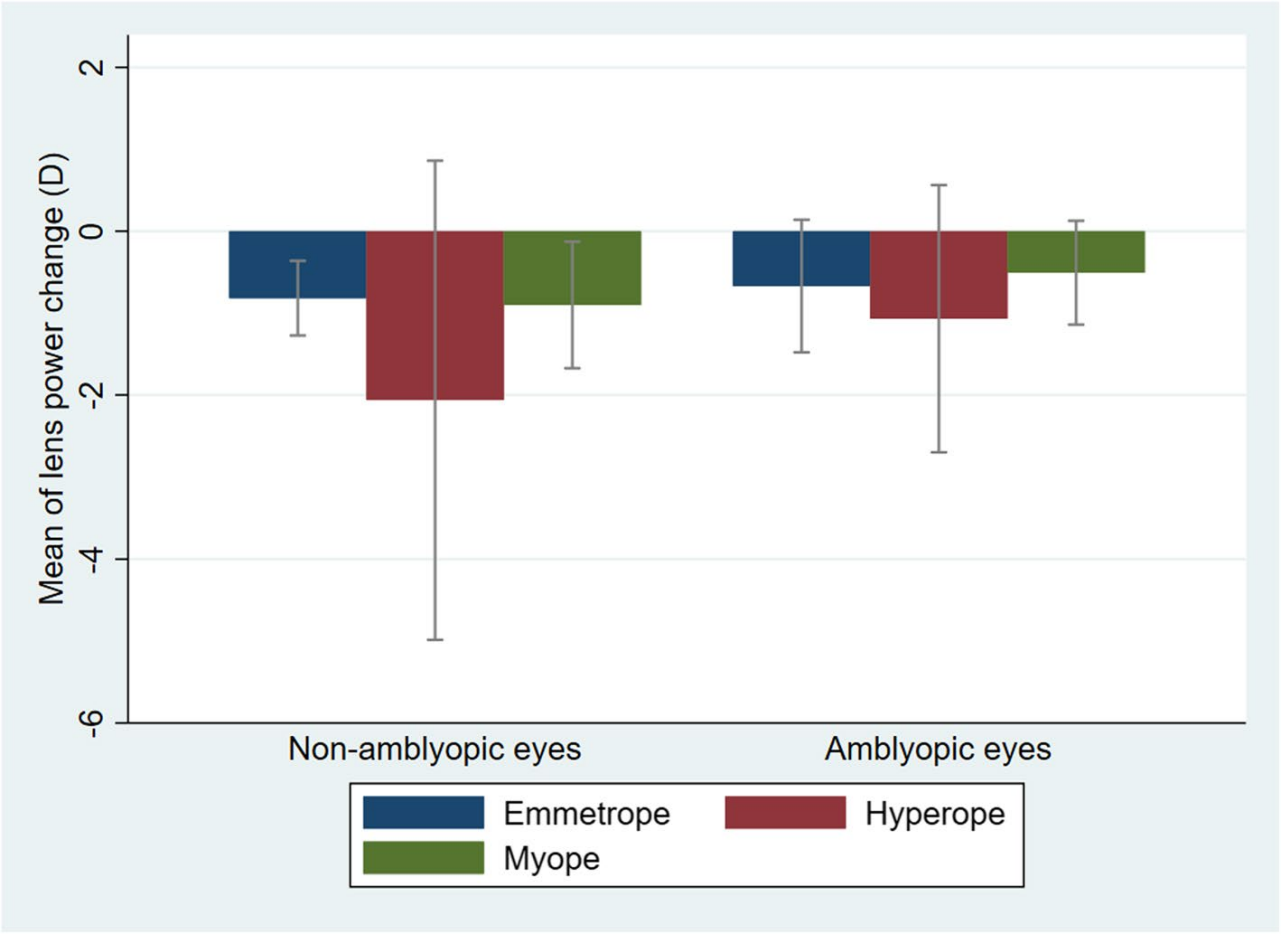
The comparison of the three-year change of lens power by refractive groups in amblyopic and non-amblyopic fellow eyes. Error bars represent 95% confidence intervals for mean changes. Negative mean change means that lens power decreased from phase 1 to phase 2 of the study

Among the 32 students with unilateral amblyopia, 7 (21.9%) were emmetropic, 9 (28.1%) had hyperopia, and 16 (50.0%) had myopia. In non-amblyopic fellow eyes, these proportions were 34.4%, 25.0%, and 40.6%, respectively. Figure 2 shows no significant differences in the mean changes in lens power between amblyopic and non-amblyopic eyes in the refractive groups.

## Discussion

Amblyopia is a neurodevelopmental disorder of vision arising from abnormal visual experience during critical periods of visual development in early childhood. It is a common cause of visual impairment, estimated to impact 1–5% of children worldwide [7]. While traditionally defined by reduced visual acuity and binocular dysfunction, emerging research underscores the structural changes in ocular growth that accompany amblyopia [13, 30]. The present large-scale study provides valuable insights into the ocular biometric changes of amblyopic eyes compared to non-amblyopic eyes over 3 years.

Prior population-based studies have reported differences in axial length, anterior chamber depth, and other ocular biometric parameters between amblyopic and non-amblyopic eyes [13, 31–33]. The significant disparities observed in key biometric parameters between amblyopic and non-amblyopic eyes in the present study further support the association between amblyopia and altered ocular growth patterns. The present study revealed greater axial elongation in amblyopic versus non-amblyopic eyes over time. Excessive axial length is implicated in myopia development and progression [33]. This exaggerated growth may relate to abnormal visual experiences disrupting emmetropization signaling [34]. The findings of the present study diverge from those of Ghasempour et al., who, in a cross-sectional study, reported longer axial length in non-amblyopic eyes [35]. The discrepancies may stem from differing study designs and lack of longitudinal follow-up. The evidence supports altered axial length elongation in amblyopia, but the nuances require further study.

Beyond axial length, understanding changes in other ocular dimensions is imperative, as subtle variations can substantially impact refraction [36]. The present study’s findings revealed differences in anterior chamber depth and vitreous chamber depth changes between amblyopic and non-amblyopic eyes. There were no significant differences in anterior chamber depth between amblyopic and non-amblyopic eyes. Additionally, there was no change in anterior chamber depth in amblyopic eyes after three years. Anterior chamber depth was similar between amblyopic and fellow eyes in another study [30]. Also, a greater increase was observed in the vitreous chamber depth of the amblyopic eyes compared to the non-amblyopic eyes. Since the vitreous chamber comprises the largest eye segment, its excessive elongation likely underlies the increased axial length seen in amblyopia [13]. Investigations into vitreous chamber depth in amblyopia remain limited, and the exact mechanisms underlying these changes require further exploration.

This study provides insight into the role the cornea might have in amblyopia, which has long been debated. The findings showed minimal alterations in corneal curvature and central corneal thickness in the amblyopic and non-amblyopic eyes of children with unilateral amblyopia over time. The radii of curvature increased significantly in eyes with amblyopia, while remaining stable in eyes without amblyopia. This suggests corneal parameters may not be substantially impacted in amblyopia after early infancy when the cornea is most malleable. Nonetheless, a major limitation in interpreting the keratometry and pachymetry findings in the present study is the lack of stratification by the amblyopia subtype.

Both amblyopic and non-amblyopic eyes displayed a significant decrease in lens power. This decrease in amblyopic eyes was not significantly different from non-amblyopic eyes. However, when comparing amblyopic eyes with their non-amblyopic fellow eyes, a greater decrease was observed in the non-amblyopic eyes. This decline in lens power indicates potential alterations in the refractive characteristics of the eyes over time. More studies with a larger sample size and longer follow-up time are needed to better understand these changes.

The findings of the present study showed that refractive changes in amblyopic and non-amblyopic eyes were similar, which contrasted with some studies that reported altered emmetropization in amblyopia [37]. Current evidence suggests that amblyopia may have an impact but does not completely hamper emmetropization.

The structural changes may represent the arrest of ocular maturation and disrupted emmetropization in amblyopia. Elucidating these growth patterns and their functional implications will be key in refining therapies to optimize visual outcomes.

The findings of the present study advocate for a more comprehensive characterization of amblyopia that looks beyond visual acuity to appreciate the broader impacts on ocular development. It should also be noted that some differences in ocular biometrics between amblyopic and non-amblyopic eyes may not be clinically significant. Large-scale longitudinal studies with diverse populations and longer follow-up periods are warranted to further unravel these changes across amblyopia subtypes and severities.

The present study had some specific limitations. The most important limitations were a relatively small number of amblyopic eyes compared to non-amblyopic eyes, lack of stratified analyses by type of amblyopia (strabismic vs. anisometropic vs. combined mechanism amblyopia), and inability to control for treatment effects during the follow-up period that could impact outcomes. Additionally, we acknowledge that the mean age of our participants was beyond the generally accepted critical period for amblyopia development. As a result, the observed changes may not fully reflect ocular growth patterns during this critical stage of amblyopia development. However, some of the most important strengths of the current research were the high participation rate in the second phase, the study design, and the use of cycloplegic refraction. A high sample size in the non-amblyopic group also increased the study power to some extent.

## Conclusion

This study highlights distinct differences in ocular growth patterns between amblyopic and non-amblyopic eyes, with amblyopic eyes exhibiting greater axial elongation and more pronounced changes in vitreous chamber depth and keratometry. These findings provide valuable insights into the impact of amblyopia on ocular development and its underlying mechanisms. Understanding these differences may aid in developing targeted screening and treatment strategies. Further research is needed to explore how these biometric changes can inform clinical practice and improve the diagnosis and management of amblyopia.

## Data Availability

The datasets used and/or analysed during the current study are available from the corresponding author on reasonable request.

## Abbreviations

SSCECS: Shahroud Schoolchildren Eye Cohort Study
UDVA: Uncorrected distance visual acuity
CDVA: Best-corrected distance visual acuity
BMI: Body mass index
D: Diopter

## References

[1] Sánchez-González MC, Palomo‐Carrión R, De‐Hita‐Cantalejo C, Romero‐Galisteo RP, Gutiérrez‐Sánchez E, Pinero‐Pinto E. Visual system and motor development in children: a systematic review. Acta Ophthalmol. 2022;100(7):e1356-69. 35118800 10.1111/aos.15111 PMC9790241

[2] Atkinson J, Braddick O. Visual development. Handbook Clin Neurol. 2020;173:121–42 Elsevier. 10.1016/B978-0-444-64150-2.00013-7 32958168

[3] West S, Williams C. Amblyopia. BMJ. Clin Evid. 2011;2011:0709. PMC3275294 21714945

[4] Holmes JM, Clarke MP. Amblyopia. Lancet. 2006;367(9519):1343–51. 16631913 10.1016/S0140-6736(06)68581-4

[5] Richardson SR, Wright CM, Hrisos S, Buck D, Clarke MP. Stereoacuity in unilateral visual impairment detected at preschool screening: outcomes from a randomized controlled trial. Investig Ophthalmol Vis Sci. 2005;46(1):150–4. 15623768 10.1167/iovs.04-0672

[6] Alrasheed SH, Aldakhil S. Childhood amblyopia: a systematic review of recent management options. Saudi J Ophthalmol. 2024;38(3):201–13. 39465021 10.4103/sjopt.sjopt_212_23 PMC11503980

[7] Hashemi H, Pakzad R, Yekta A, Bostamzad P, Aghamirsalim M, Sardari S, et al. Global and regional estimates of prevalence of amblyopia: a systematic review and meta-analysis. Strabismus. 2018;26(4):168–83. 30059649 10.1080/09273972.2018.1500618

[8] Group PEDI. A randomized trial of atropine vs patching for treatment of moderate amblyopia in children. Arch Ophthalmol. 2002;120(3):268–78. 11879129 10.1001/archopht.120.3.268

[9] Stewart CE, Moseley MJ, Georgiou P, Fielder AR. Occlusion dose monitoring in amblyopia therapy: status, insights, and future directions. J Am Association Pediatr Ophthalmol Strabismus. 2017;21(5):402–6. 10.1016/j.jaapos.2017.06.018 28890077

[10] Semeraro F, Forbice E, Nascimbeni G, Cillino S, Bonfiglio VME, Filippelli ME, et al. Ocular refraction at birth and its development during the first year of life in a large cohort of babies in a single center in Northern Italy. Front Pead. 2020;7:539. 10.3389/fped.2019.00539 PMC7001530 32083036

[11] Troilo D, Wallman J. The regulation of eye growth and refractive state: an experimental study of emmetropization. Vision Res. 1991;31(7–8):1237–50. 1891815 10.1016/0042-6989(91)90048-a

[12] Mutti DO, Mitchell GL, Jones LA, Friedman NE, Frane SL, Lin WK, et al. Axial growth and changes in lenticular and corneal power during emmetropization in infants. Investig Ophthalmol Vis Sci. 2005;46(9):3074–80. 16123404 10.1167/iovs.04-1040

[13] Cass K, Tromans C. A biometric investigation of ocular components in amblyopia. Ophthalmic Physiol Opt. 2008;28(5):429–40. 18761480 10.1111/j.1475-1313.2008.00585.x

[14] Demircan S, Gokce G, Yuvaci I, Ataş M, Başkan B, Zararsiz G. The assessment of anterior and posterior ocular structures in hyperopic anisometropic amblyopia. Med Sci Monitor Int Med J Experimental Clin Res. 2015;21:1181. 10.12659/MSM.893979 PMC4422113 25910432

[15] Hashemi H, Pakzad R, Khabazkhoob M, Yekta A, Emamian MH, Fotouhi A. Ocular biometrics as a function of age, gender, height, weight, and its association with spherical equivalent in children. Eur J Ophthalmol. 2021;31(2):688–97. 32103688 10.1177/1120672120908722

[16] Noya-Padin V, Nores-Palmas N, Garcia-Queiruga J, Giraldez MJ, Pena-Verdeal H, Yebra-Pimentel E, editors. Associations between ocular biometry, refractive error, and body characteristics. Photonics. 2024;11(2):165.

[17] Mutti DO, Sinnott LT, Mitchell GL, Jordan LA, Friedman NE, Frane SL, et al. Ocular component development during infancy and early childhood. Optometry Vis Sci. 2018;95(11):976. 10.1097/OPX.0000000000001296 PMC6212316 30339640

[18] Baisakhiya S, Singh S, Garg P. Are the biometric parameters predictors of refractive and accommodative status of the eye? Natl J Physiol Pharm Pharmacol. 1970;6(6):550.

[19] Robaei D, Rose KA, Ojaimi E, Kifley A, Martin FJ, Mitchell P. Causes and associations of amblyopia in a population-based sample of 6-year-old Australian children. Arch Ophthalmol. 2006;124(6):878–84. 16769842 10.1001/archopht.124.6.878

[20] Li S-M, Liu L-R, Li S-Y, Ji Y-Z, Fu J, Wang Y, et al. Design, methodology and baseline data of a school-based cohort study in Central China: the Anyang Childhood Eye Study. Ophthalmic Epidemiol. 2013;20(6):348–59. 24160405 10.3109/09286586.2013.842596

[21] Sng CC, Lin X-Y, Gazzard G, Chang B, Dirani M, Lim L, et al. Change in peripheral refraction over time in Singapore Chinese children. Investig Ophthalmol Vis Sci. 2011;52(11):7880–7. 21873673 10.1167/iovs.11-7290

[22] Mallen EA, Gammoh Y, Al-Bdour M, Sayegh FN. Refractive error and ocular biometry in Jordanian adults. Ophthalmic Physiol Opt. 2005;25(4):302–9. 15953114 10.1111/j.1475-1313.2005.00306.x

[23] Emamian MH, Hashemi H, Khabazkhoob M, Malihi S, Fotouhi A. Cohort profile: Shahroud Schoolchildren Eye Cohort Study (SSCECS). Int J Epidemiol. 2019;48(1):27-f. 30534958 10.1093/ije/dyy250

[24] Group M-ePEDS. Prevalence of amblyopia and strabismus in African American and hispanic children ages 6 to 72 months: the multi-ethnic Pediatric Eye Disease Study. Ophthalmology. 2008;115(7):1229–36. 17953989 10.1016/j.ophtha.2007.08.001 PMC4839485

[25] Repka MX, Holmes JM. Lessons from the amblyopia treatment studies. Ophthalmology. 2012;119(4):657–8. 22472249 10.1016/j.ophtha.2011.12.003

[26] Chen AM, Cotter SA. The amblyopia treatment studies: implications for clinical practice. Adv Ophthalmol Optometry. 2016;1(1):287–305. 10.1016/j.yaoo.2016.03.007 PMC5396957 28435934

[27] Wallace DK. Evidence-based amblyopia treatment: results of PEDIG studies. Am Orthopt J. 2007;57(1):48–55.

[28] Cruysberg LP, Doors M, Verbakel F, Berendschot TT, De Brabander J, Nuijts RM. Evaluation of the Lenstar LS 900 non-contact biometer. Br J Ophthalmol. 2010;94(1):106–10. 19692383 10.1136/bjo.2009.161729

[29] Beenett A. A method of determining the equivalent powers of the eye and its crystalline lens without resort to phakometry. Ophthalmic Physiol Opt. 1988;8(1):53–9. 3047630 10.1016/0275-5408(88)90089-0

[30] Debert I, Polati M, Jesus DLd, Souza ECS, Alves MR. Biometric relationships of ocular components in esotropic amblyopia. Arq Bras Oftalmol. 2012;75:38–42. 22552416

[31] Khan AO. The relationship of axial length to cycloplegic refraction and keratometry in amblyopic eyes of hyperopic children. J Am Assoc Pediatr Ophthalmol Strabismus. 2012;16(1):46–8. 10.1016/j.jaapos.2011.10.006 22370665

[32] Cass K. Ocular components in amblyopia: a biometric investigation: the University of Manchester (United Kingdom); 2005.

[33] Xie R, Zhou XT, Lu F, Chen M, Xue A, Chen S, et al. Correlation between myopia and major biometric parameters of the eye: a retrospective clinical study. Optom Vis Sci. 2009;86(5):E503-8. 19349927 10.1097/OPX.0b013e31819f9bc5

[34] Wallman J, Winawer J. Homeostasis of eye growth and the question of myopia. Neuron. 2004;43(4):447–68. 15312645 10.1016/j.neuron.2004.08.008

[35] Ghasempour M, Khorrami-Nejad M, Akbari MR, Amiri MA. The effect of different amblyopia treatment protocols on axial length of non-amblyopic eyes in anisohyperopic patients. J Curr Ophthalmol. 2019;31(2):201–5. 31317100 10.1016/j.joco.2018.09.003 PMC6611861

[36] Atchison DA, Jones CE, Schmid KL, Pritchard N, Pope JM, Strugnell WE, et al. Eye shape in emmetropia and myopia. Investig Ophthalmol Vis Sci. 2004;45(10):3380–6. 15452039 10.1167/iovs.04-0292

[37] Chen Y, Zuo J, Xiong Y, Yu X, Wei L, Luo Y, et al. Refraction development in anisometropic amblyopia with patching therapy. Front Med (Lausanne). 2022;9:959085. 36330057 10.3389/fmed.2022.959085 PMC9623012

